# An investigation of perceived risk dimensions in acceptability of shared autonomous vehicles, a mediation-moderation analysis

**DOI:** 10.1038/s41598-024-74024-0

**Published:** 2024-10-07

**Authors:** Mohammadhossein Abbasi, Amir Reza Mamdoohi, Francesco Ciari, Grzegorz Sierpiński

**Affiliations:** 1 Faculty of Civil and Environmental Engineering, Tarbiat Modares University, Tehran, Iran; 2 Department of Civil, Geological and Mining Engineering, Polytechnique Montréal, Montreal, QC H3T 1J4 Canada; 3 Department of Transport Systems, Traffic Engineering and Logistics, Faculty of Transport and Aviation Engineering, Silesian University of Technology, Gliwice, 44-100 Poland

**Keywords:** Shared autonomous vehicle, Ride-sharing, Intention to use, Perceived risk dimensions, Generation, Trust, Civil engineering, Statistics

## Abstract

The integration of shared and autonomous mobility has led to the emergence of shared autonomous vehicles with ride-sharing services (SAVWRS), which have the potential to significantly reduce private car usage and promote sustainable transportation. Despite numerous studies on this topic, there is still no research examining the impact of all dimensions of perceived risk theory on usage intention. Therefore, we aim to investigate these relationships and gain deeper insights by examining the mediating effect of trust and the moderating effect of generation (Millennials vs. Baby Boomers) to address potential disparities across generations. To gather insights, we design an online survey that was completed by a random sample of 723 individuals in 2021. The estimation results of the structural equation model reveal that all perceived risk dimensions (social, performance, time, physical, security, and financial risks, in descending order) are negatively related to consumers’ intention. Additionally, trust fully mediates the relationships between performance, physical, financial, and security risks and usage intention, whereas it partially mediates the relationships between social and time risks and the intention to use. Furthermore, moderation analysis revealed that Millennials are less concerned about most dimensions of perceived risk theory, except for social and time risks. In conclusion, our study contributes to a deeper understanding of the complex relationships between perceived risk dimensions, trust, and usage intention in SAVWRS. Our findings suggest that policymakers and industry stakeholders should consider strategies to address these concerns to promote widespread acceptance of SAVWRS.

## Introduction

With the advent of autonomous vehicles, new transportation services are being provided under the umbrella of autonomous mobility services. Human-driven vehicles are likely to be replaced by these services in the near future^1^. A shift is expected from driver-assistance to driver-replacement technologies in fully autonomous vehicles (FAVs). FAVs are projected to capture 50% of the new vehicle market by 2050^2^. It is anticipated that various aspects of human life will be affected by the emergence of FAVs, such as significant reductions in injuries and fatal accidents^3^, private car ownership^4^, and fossil fuel consumption^5^as well as improvement in network performance and traffic flow^6^, independent mobility for the elderlies, disabled, and people without driving license, and land use^7^. Moreover, little human intervention is necessary during trips, enabling more productive and efficient activities to be conducted^8^. Another advantage of FAVs is their ability to facilitate ride-sharing and offer more promising prospects by overcoming some of the biggest challenges of sharing services, such as accessibility and reliability^9^. On the other hand, collaborative consumption can make the deployment of FAVs financially feasible and viable^10,11^. Thus, ride-sharing services in FAVs could be an appealing solution for the development of a sustainable transportation system^12^. Shared autonomous vehicles with ride-sharing services (SAVWRS), as a result of integrating shared mobility and autonomous services, can improve travel efficiency and vehicle capacity utilization by effectively using empty vehicle seats without increasing the load on urban infrastructure, subsequently reducing traffic congestion in urban areas^13,14^. Increased vehicle utilization rates can also lead to a reduction in energy consumption and CO_2 _emissions^15^.

Despite the aforementioned advantages, several uncertainties are related to this technology, including high purchase costs, privacy and trust concerns, and legal and liability issues^16^. SAVWRS can collect individual information regarding users’ trips and personal characteristics, which may intrude on their privacy^17^. As noted by Liu et al., respondents expected SAVWRS to be significantly safer than traditional human-driven vehicles (four to five times safer)^18^. However, there is a possibility that this opinion may change quickly if a single incident occurs, such as the fatal incident in 2018 when an FAV struck and killed a pedestrian^19^. While this does not change the fact that FAVs are safer^20,21^, it may affect people’s perceptions. Moreover, according to a survey in the U.S., more than 80% of respondents were concerned about potential safety implications, while only 33% were concerned about their privacy^22^.

In recent years, numerous studies have explored the acceptance of FAVs and highlighted consumers’ perceived risk as a significant barrier to public acceptance, especially among individuals with different levels of experience and knowledge of FAVs^23,24^. For example, examining the intention to use SAVWRS among Baby Boomers (generally referred to individuals born approximately between 1946 and 1964) and Millennials (generally referred to individuals born between 1981 and 1996^25^) is pivotal for comprehending how varying generational viewpoints impact the acceptance of such innovative technologies. These two demographic cohorts embody distinct life stages and socio-cultural environments, which can result in markedly different attitudes towards advancements in transportation. Millennials, having been raised amid rapid technological progress and typically exhibiting a greater enthusiasm for new mobility options, may demonstrate a higher likelihood of embracing SAVWRS. This inclination is often attributed to their familiarity with digital platforms and a strong preference for sustainable practices. Conversely, Baby Boomers may exhibit a more cautious stance toward acceptance of SAVWRS, often shaped by considerations such as perceived safety, trust in technological innovations, and historically ingrained transportation behaviors. By investigating these generational disparities, researchers and policymakers can derive meaningful insights into the distinct motivations and apprehensions of each group, thereby facilitating the creation of customized strategies aimed at promoting the acceptance and effective implementation of SAV services. Grasping these interactions is crucial not only for improving user experiences but also for addressing broader societal issues concerning mobility, urban planning, and environmental sustainability. On the other hand, previous studies have revealed that causal links for perceived risk primarily been established between perceived risks and trust or behavioral intention^26–29^. However, different results have been obtained, raising questions regarding the impact of perceived risk^26^. This suggests that further research is required to determine how and to what extent perceived risk will influence SAVWRS acceptability through the mediating effect of trust^27^.

After critically reviewing prior studies on the acceptance of FAVs (Table 1), it becomes evident that not much attention has been given to perceived risk as a construct with multiple dimensions. The rationale for considering different dimensions of perceived risk stems from Kenesei et al.‘s finding that treating perceived risk as a one-dimensional construct leads to contradictory results^26^. Moreover, in a study by Lee et al.^30^, they pointed out that the wrong definition of perceived risk has contributed to inconclusive results in previous studies^31,32^. We have thus further modelled perceived risk into six distinct dimensions (performance risk, financial risk, social risk, security risk, physical risk, and time risk), as suggested by Paul and Tarpey^33^base on the perceived risk theory by Jacoby and Kaplan^34^. Another research gap lies in the fact that, although social risk perception has been one of the most studied factors in consumer marketing over the past decades, it is rarely explored in recent years, especially in studies related to SAVWRS acceptance^29^. Finally, it can be concluded that although perceived risk has been somewhat studied, there are still several gaps in the literature and our research contributes to the existing body of literature in the following manner. To the best of our knowledge, our research is among the first studies exploring the impact of all dimensions of perceived risk (i.e., performance risk, financial risk, social risk, security risk, physical risk, and time risk) on intention to use SAVWRS. Additionally, little attention has been given to the generational differences in the impact of perceived risk dimensions on SAVWRS acceptability. Further, most of the related literature focused on private FAVs, whereas SAVWRS have not receive much attention, despite being well-aligned with sustainable transportation development. Lastly, previous studies have primarily been conducted in developed countries, and as far as we know, there have been no studies on this topic in developing countries. Therefore, understanding how perceived risk dimensions influence potential consumers’ intentions is critical for various purposes, particularly policymaking.

**Table 1:** A brief review of previous studies incorporated the perceived risk dimensions in autonomous vehicles.

Study	Year	Moderation/mediation	Perceived risk dimension	Sample size	Model
Aboutorabi Kashani et al.^14^	2024	-	General	491	BL
Koh et al.^35^	2024	Mediation (PR→BI)	PR; SER	500	SEM
Khan et al.^36^	2024	Mediation (PR→)BI	PR; PHR; SER	2062	SEM
Staab & Liebherr^37^	2024	-	PR	435	SEM
Korkmaz et al.^38^	2023	Moderation (Age, Gender)	FR, PR	316	SEM
Orsot-dessi et al.^39^	2023	-	PR	113	SEM
Abbasi et al.^12^	2023	-	PR	743	GOL
Farzin et al.^40^	2023	-	PR	641	SEM
Waung et al.^28^	2021	Mediation (TR→BI)	SER; PR	337	SEM
Ribeiro et al.^41^	2022	-	PR	362	SEM
Benleulmi & Ramdani^42^	2022	-	PR; SER	240	SEM
Kenesei et al.^26^	2022	Mediation (TR→PR)	PR; SER	949	SEM
Zhang et al.^27^	2019	Mediation (TR→BI)	SER; PR	206	SEM
Liu et al.^43^	2019	Mediation (TR→BI)	General	441	SEM
Li et al.^44^	2019	-	General	63	ANOVA
Xu et al.^32^	2018	Mediation (TR→PR)	PR	171	SEM

BI: Behavioral intention; BL: Binary Logit; FR: Financial Risk; GOL: Generalized Ordered Logit, PHR: Physical Risk; PR: Performance Risk; SER: Security Risk; SOR: Social Risk; TIR: Time Risk; TR: Trust.

To address the research gaps and understand how to achieve widespread acceptance of SAVWRS as a sustainable innovation, this study examines the relationship between different dimensions of perceived risk and consumer behavioral intention, along with the mediating effect of trust and the moderating effect of generation. Specifically, this research seeks to answer the following questions:

1. How and to what extent do different dimensions of perceived risk influence the acceptability of SAVWRS?
2. Whether and how “Trust” mediates the relationships between different dimensions of perceived risk and usage intention of SAVWRS?
3. Whether and how “Generation” moderate the causal pathways between different dimensions of perceived risk and the acceptability of SAVWRS?

By addressing these questions, this study aims to provide a more comprehensive understanding of the factors affecting the acceptance of SAVWRS. The outcomes are expected to reveal critical insights into consumer behavior, highlighting the importance of trust in mitigating perceived risks and the role of generational differences in shaping attitudes toward this innovative technology.

The rest of the paper is structured in the following order. Based on the literature review, we present a theoretical framework and formulate research hypotheses in “Theoretical framework and research hypotheses” section. In “Research methodology” section discusses the methodology, development of measurement instruments and the management of survey. The research is then focused on the analysis of data and the presentation of results in “Data analysis and result” section. As a conclusion, “Discussion” discusses both theoretical and practical implications as well as research limitations and suggestions for further research.

## Theoretical framework and research hypotheses

### Perceived risk and its dimensions

Jacoby and Kaplan^34^defined perceived risk as consumer perceptions of uncertainty and negative consequences that may arise from purchasing a product or service. Based on the definition provided by Schiffman and Kanuk, consumers are more likely to perceive risk when they cannot predict the consequences of their purchase/use decisions^45^. According to Jacoby and Kaplan^34^, perceived risks can be categorized into five dimensions: financial risk, performance risk, physical risk, security risk, and social risk^34^. In 1975, Paul and Tarpey suggested that time risk should also be included as a component of perceived risk^33^. This theory has a wide range of application in different topics; however, it has been attempted to customized the contents within the scope of SAVWRS. As can be seen, most of prior studies mentioned in Table 1, has considered only a limited number of these components. These components as well as their corresponding hypotheses that has been formulated will be presented in the following subsections.

#### Financial risk

Financial risk, as one of the dimensions of perceived risk theory, may be a concern for potential consumers if believe they will need to pay more than the purchase price or if taxes will be incurred when they purchase the product^46^. Financial risk has been considered in various research studies such as the purchase intention of electric vehicles^47^, intention to visit edible insect and live seafood restaurants^48^, electronic word-of-mouth adoption^49^, internet-banking adoption^50^, and continuance intention toward bike-sharing services^51^. However, there are a few studies, like Korkmaz et al.^38^, that consider the influence of financial risk on the usage intention of FAVs, highlighting the need for further exploration as suggested by Ho et al.^29^, since a consumer may be reluctant to use SAVWRS if there is a risk of financial loss related to an insufficient level of maintenance and support. In case of SAVWRS, it is anticipated that consumers often prioritize the initial costs of utilizing SAVWRS, such as subscription fees or per-ride charges, while neglecting additional expenses like maintenance, insurance, and taxes. If these supplementary costs are perceived as substantial, they may discourage potential users, thereby heightening the financial risk associated with adopting SAVs. Furthermore, many SAV services are expected to implement dynamic pricing based on factors such as demand, time of day, or distance traveled. This variability can raise concerns about financial risk, as consumers might fear that costs could surpass their budget, particularly during peak periods. Additionally, if local governments impose specific taxes or fees on SAVWRS usage, consumers may view this as an extra financial burden, potentially influencing their willingness to use these services by perceiving the total cost as outweighing the benefits. Therefore, based on the findings of the previous studies mentioned and perceived risk theory, it can be hypothesized that financial risk negatively impacts the usage intention of potential consumers, leading to the formulation of the following hypothesis.

*H1: Perceived financial risk is negatively related to consumers’ usage intention of SAVWRS.*

#### Performance risk

Individuals who believe that a product they intend to use will not perform its primary function experience performance risk, which can lead to feelings of remorse and dissatisfaction if the product fails^46^. For instance, in the context of SAVWRS, consumers may express concerns about potential technical failures, such as software glitches or hardware malfunctions, which they perceive as compromising safety and vehicle functionality, thereby contributing to significant performance risk. Additionally, apprehensions about the reliability of autonomous technology, particularly regarding a vehicle’s ability to navigate safely and efficiently, further exacerbate these performance risks. Incidents involving accidents or malfunctions in autonomous vehicles amplify these concerns, resulting in increased hesitation among potential users to use SAV services. This dimension of perceived risk theory has been extensively studied in prior research, including works by Koh et al.^35^, Khan et al.^36^, Staab and Liebherr^37^, and others mentioned in Table 1, all of which found a negative correlation between performance risk and usage intention of FAVs. Based on perceived risk theory and the findings of previous studies, it is expected that performance risk is negatively associated with usage intention. Therefore, the following hypothesis is proposed.

*H2: Perceived performance risk is negatively related to consumers’ usage intention of SAVWRS.*

#### Physical risk

Physical risk refers to the possibility that a product or service may cause physical or mental harm to a person, their family members, or others who use the product or service. In the context of this study, physical risk refers to customers’ concerns about any physical or mental risks that may arise from using SAVWRS, due to the absence of a driver in the vehicle or potential harassment from strangers. In other words, consumers may have concerns about potential malfunctions in autonomous technology that could result in collisions, causing physical harm to passengers, pedestrians, or other road users. High-profile accidents involving autonomous vehicles can intensify these fears. Additionally, the prospect of traveling in a vehicle without a human driver can provoke anxiety in some individuals. Concerns regarding safety and control may lead to mental distress, thereby affecting their willingness to utilize SAV services. Physical risk has been considered in various research studies such as purchase intention for electric vehicles^47^, consumers’ purchase intention toward the retailers’ private labels^52^, air travel satisfaction and repurchase intention^53^, adoption of shared accommodation and office services^54^, intention to discontinue use of ride-hailing services^55^, and revisit intention for tourism^56^. However, there are a few studies, like Khan et al.^36^, that consider the influence of physical risk on usage intention of FAVs, highlighting the need for further exploration as suggested by Ho et al.^29^. All of the aforementioned studies found that physical risk has a negative impact on usage intention. Based on perceived risk theory and the findings of previous studies, physical risk is negatively associated with usage intention. Therefore, the following hypothesis is proposed.

*H3: Perceived physical risk is negatively related to consumers’ usage intention of SAVWRS.*

#### Security risk

Security risk is another issue that has garnered the attention of consumers^2,57,58^. A potential concern arises from the possibility of unauthorized access to travel or behavioral data by government entities, vehicle manufacturers, and insurance companies, or from the potential misuse or hacking of this data^27^. In a more-detailed explanation, SAVWRSs may collect individual facial images for authentication or personalized services, track passengers’ routines and working hours, aggregate traffic data that could reveal sensitive information about communities or individuals, and monitor movement patterns that might disclose personal habits or preferences. Additionally, sentry modes in parked SAVWRSs record surroundings for security purposes, and these recordings may contain personally identifiable information without consent. This dimension of perceived risk theory has been frequently investigated in previous studies, such as those by Koh et al.^35^, Khan et al.^36^, Waung et al.^28^, Kenesei et al.^26^, Zhang et al.^27^, and Benleulmi and Ramdani^42^, all of which found a negative correlation between security risk and the usage intention of FAVs. Based on the perceived risk theory and findings of previous studies, the following hypothesis is proposed.

*H4: Perceived security risk is negatively related to consumers’ usage intention of SAVWRS.*

#### Social risk

Social risk refers to the perception that a user may lose status within their social group due to the use or purchase of a particular good or service^46,59^. Notably, an individual’s social position and participation in ride-sharing services may be undervalued if the crowd perceives ride-sharing as inferior^60^. For those who are concerned about their societal standing and how others perceive them, using shared services may pose a challenge compared to driving their own vehicles^61^. In other words, using SAVs, which are often viewed as a shared or communal resource, may lead some consumers to feel that they are sacrificing their social status. This perception can deter potential users who prioritize personal ownership as a symbol of achievement. If ride-sharing services are seen as inferior or less desirable compared to traditional car ownership, individuals may fear being judged by their peers. This stigma can create a barrier to adopting SAVs, as users may worry about how they will be perceived by friends, family, and colleagues. Social risk has been examined in various research studies, such as those on branding^62^, marketing^63^, online merchant selection^64^, visiting unconventional restaurants^48^, and car sharing systems^61^. However, there are currently no studies that consider the influence of social risk on the usage intention of FAVs, highlighting the need for further exploration as suggested by Ho et al.^29^, since a consumer may be reluctant to use SAVWRS if there is a risk of status loss related to an insufficient level of prestige. Investigation of the aforementioned studies alongside perceived risk theory indicates that there is a negative association between social risk and usage intention of SAVWRS. Based on the findings of previous studies, the following hypothesis is proposed.

*H5: Perceived social risk is negatively related to consumers’ usage intention of SAVWRS.*

#### Time risk

Time risk refers to the potential loss of time caused by a wrong purchase or use decision that fails to yield the expected results and necessitates replacement^46^. It is associated with lost convenience, lost time, or waste effort in getting a service redone^65^. In the context of shared mobility, time risk specifically relates to meeting the consumer’s expected timeframe for reaching their destination^66^. More specifically, Consumers anticipate that SAVWRS will deliver reliable and timely services. Delays or inconsistent arrival times can lead to frustration and a heightened perception of time risk, particularly for individuals with strict schedules or commitments, such as work or appointments. The time spent waiting for an SAVWRS can exacerbate this concern. If consumers frequently experience longer-than-expected wait times, they may be discouraged from using the service, fearing it will waste their time. In addition, the efficiency of the vehicle’s navigation system is crucial in mitigating time risk. If consumers believe that the SAV may take longer routes or struggle with traffic management, they may hesitate to rely on the service, fearing delays that could disrupt their schedules. Additionally, if the booking process for an SAV is perceived as complicated or time-consuming, it can further increase time risk. Users may prefer traditional transportation methods that they perceive as quicker and more straightforward, leading to reluctance in adopting SAVs. Time risk has received less attention in studies within the scope of FAV systems. Investigations of perceived risk theory indicate that there is a negative association between time risk and usage intention of SAVWRS. Based on this theory, the following hypothesis is proposed.

*H6: Perceived time risk is negatively related to consumers’ usage intention of SAVWRS.*

### Trust mediating effect

There is a substantial body of research that confirms the critical role of perceived risk; however, the nature of the relationship between perceived risk, trust, and consumer acceptance remains controversial^67^. The most debated topic has been the directionality of effects and antecedents concerning trust and risk^27^. A higher level of trust is associated with a reduction in perceived risk over time, as in some studies, trust has been viewed as a significant antecedent to risk^43,68^. A model of AV acceptance has been developed by Choi and Ji that takes this relationship into account^31^. However, others believe that, according to Mitchell^69^, trust can become operational only when both parties are exposed to some level of risk and are somewhat vulnerable^69^. Empirical studies have also supported this relationship^70^. We consider perceived risk as a predictor of initial trust, as trust is largely established through knowledge of AV benefits and risks^71^. To put it another way, trust cannot be established without a certain level of risk. Finally, it was hypothesized that perceived risk would have no direct effect on usage intention in this study for two reasons. Firstly, a recent meta-analysis examining the relationships among trust, risk, and acceptance indicated that eliminating the causal path between perceived risk and usage intention was associated with enhanced model goodness of fit. According to Choi and Ji^31^and Xu et al.^32^, perceived risk has no direct impact on usage intention in their AV acceptance models^32^. Accordingly, we found that initial trust mediated the effect of perceived risk on usage intention in our model. Therefore, the following hypotheses have been formulated.

*H7: Trust will mediate the relationship between perceived financial risk and consumers’ usage intention of SAVWRS (FR→TR→BI).*

*H8: Trust will mediate the relationship between perceived performance risk and consumers’ usage intention of SAVWRS (PR→TR→BI).*

*H9: Trust will mediate the relationship between perceived physical risk and consumers’ usage intention of SAVWRS (PHR→TR→BI).*

*H10: Trust will mediate the relationship between perceived security risk and consumers’ usage intention of SAVWRS (SER→TR→BI).*

*H11: Trust will mediate the relationship between perceived social risk and consumers’ usage intention of SAVWRS (SOR→TR→BI).*

*H12: Trust will mediate the relationship between perceived time risk and consumers’ usage intention of SAVWRS (TIR→TR→BI).*

*H13: Trust is positively correlated with consumers’ usage intention of SAVWRS (TR→BI).*

### Generation moderating effect

In this study, we consider the moderating effect of generation on the relationships discussed. It is well established that millennials have grown up with technology and are more familiar with it than their older counterparts. Additionally, millennials are among the most active generations on social media^72^. Further, in prior studies^73–76^, they have concluded that it is more likely that millennials adopt the FAVs and are less concerned about the risks associated with using such technologies. Given their propensity for innovativeness^77^, we can expect that millennials will be more inclined to use and trust SAVWRS compared to baby boomers in the future. Therefore, the following hypotheses have been formulated.

*H14-20: There is a significant moderating effect of generation in relationship between perceived risk and trust*,* and trust and usage intention.*

**Fig. 1:**
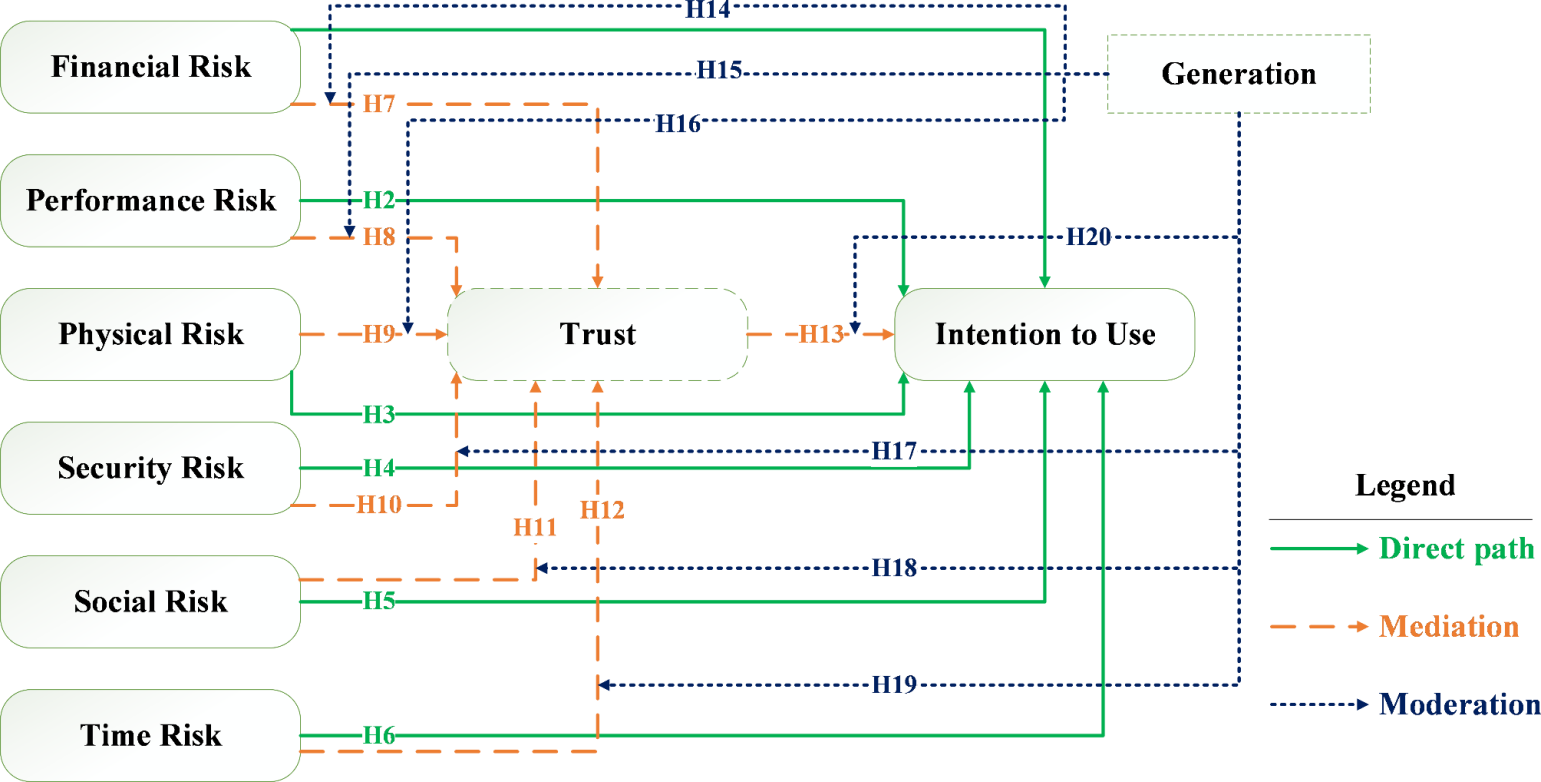
Research conceptual model and hypotheses.

Upon reviewing the aforementioned studies, it is evident that most prior research addressing the acceptance of FAVs did not distinguish between different dimensions of perceived risk. Therefore, based on our research questions and hypotheses, as well as the gaps identified in prior studies, which have been thoroughly discussed in the “Introduction” section, we have developed a conceptual model shown in Fig. 1. Furthermore, the mediating effect of trust and the moderating effect of generation have been investigated.

## Research methodology

### Structural equation modelling

SEM is a statistical methodology that allows researchers to explore and analyze complex relationships among independent variables (IVs) and dependent variables (DVs). Both IVs and DVs can be either continuous or discrete. SEM enables researchers to investigate causal pathways and test hypotheses about latent constructs. It has been composed of two sub-models including measurement and structural models. The later represents the hypothesized causal relationships among latent variables and these relationships are often expressed using equations. However, the former describes the connection between latent constructs (ξ and η) and their observed indicators (Xi or Yi). Factor loadings quantify the strength of these relationships. γ and β represent the relationships between exogenous and endogenous variables in the structural model, whereas in the measurement model, factor loadings are represented by λ. The error term in the structural model is denoted by ζ, while in the measurement model, error terms for measuring endogenous and exogenous latent constructs are ε and δ, respectively^78^. Equation (1) to (3) illustrate the relationships among the variables within an SEM model.

1 $$\eta = {\rm B}\eta + \Gamma \xi + \zeta$$

2 $$X = \Lambda _{x} \xi + \delta$$

3 $$Y = \Lambda _{y} \eta + \varepsilon$$

Mediation and moderation analyses are essential because they help us gain a deeper understanding of how different variables relate to one another. By exploring these techniques, we can not only identify whether two variables are connected, but also clarify how this connection works and the specific situations in which it occurs^79^.

Mediation focuses on the processes that explain how one IV affects another DV through intermediary factors known as mediators. Understanding these paths is vital for fully grasping complex issues. On the other hand, moderation looks at the various circumstances that can alter the strength and/or direction of the relationship between an IVt and a DV. This perspective is crucial for recognizing how external factors—like demographic or specific contexts—can either strengthen or weaken the relationships we are studying. Together, these concepts contribute significantly to our overall understanding of how various factors interact in our research^79^.

### Measurement

The empirical data for this study were collected using a self-administered questionnaire. After reviewing pertinent technical literature, examining prior validated questionnaires, and consulting experts in the field of FAVs, we designed a questionnaire. Items were adapted from the literature (Table 3) and partly developed/customized for our specific target population, thereby justifying their appropriateness for quantifying latent variables in our research. Due to the pandemic, we conducted an online survey in Tehran for two months starting in July 2021. To ensure clarity and comprehensibility, first, we administered a pilot study with a sample of 30 questionnaires. Based on pilot questionnaire feedback, we modified several questions to improve the clarity and comprehensibility of our questionnaire. For the purpose of collecting data, we used Epoll, an online survey platform which is well-known in Iran. After designing our questionnaire, based on our prior experience in other research projects, we used Epoll because of its user-friendly interface, robust design capabilities, Farsi language capabilities, and popularity. Epoll’s features allowed us to create various question types tailored to our research needs resulting in a reliable data base collection mechanism. Concerning the randomness of respondents, the invitation link of questionnaire was distributed through administrators of most-widely-used channels and groups in Telegram and we did not focus on any particular group. Respondents could browse our survey on Epoll platform in their cellphone and respond to the survey. Regarding the constraints for participation in the survey, only citizens of Tehran who were 18 years of age or older were eligible to participate. Additionally, we employed a stratified quota sampling technique to ensure comprehensive representation from each geographical district. By utilizing the most recent census data, we were able to ascertain the population size of each district, which enabled us to establish the targeted quotas reflecting the demographic characteristics across Tehran’s districts. Moreover, we established several criteria to check the validity of responses like the reverse question test, answer duration test, and logical consistency assessment of feedback. Duplicate responses were identified and deleted using the participants’ IP addresses. Finally, since some respondents might rush through questionnaires just to collect the reward, we did not offer any monetary prizes, as many studies have found that the corresponding disadvantages significantly outweigh their advantages^80^.

Three sections were included in the questionnaire. In the first section, after describing the survey objectives and ensuring that individual responses would remain confidential and anonymous, respondents were asked about their driving experience, accident history and severity in the last five years, their experiences with existing shared mobility services (such as carsharing), and their preferred modes of transportation. In the second section, a video introduction accompanied by a textual description of SAVWRS was provided to inform participants about the service. Following the review of all provided instructions, participants were instructed to complete the questionnaire. Following the introduction, a question asked respondents whether they had ever heard of SAVWRS before this survey and their familiarity level (ranging from 1: first time hearing of SAVWRS to 4: Completely familiar). We then presented scales measuring the psychological constructs in the proposed model (see Fig. 1). The items were derived from validated measurement scales based on a literature review. Some measurement items were modified to fit the context of the SAVWRS study. After a review by a group of experts and a pilot study involving 30 participants, further modifications were made. All items were measured on a five-point Likert scale ranging from “strongly disagree (= 1)” to “strongly agree (= 5).” Finally, the third section captured socioeconomic characteristics, including gender, age, marital status, education level, income, household size, and private car ownership.

## Data analysis and result

### Participant

After removing invalid and incomplete responses, a total of 723 valid responses from the population of Tehran were used for further analysis. Table 2 provides a descriptive analysis of the socioeconomic characteristics of the respondents, showing a balanced proportion of men and women (50.8% vs. 49.2%). Regarding marital status, 411 respondents (56.2%) were single, while the remaining 154 (24.9%) were married. The survey sample included 240 respondents with a master’s degree (33.2%). Additionally, 373 respondents (51.6%) reported owning only one private car in their household. A total of 384 respondents (53.1%) had never heard of SAVWRS before the survey, indicating a lack of familiarity with the technology. Moreover, 50% of the participants reported that their income was average. Furthermore, approximately 67% of respondents belonged to households with three or four members. Finally, the majority of respondents (82.2%) possessed a driving license, while individuals with varying levels of driving experience were also considered.

**Table 2:** Descriptive analysis of individuals’ and households’ characteristics.

Characteristic	Item	Frequency		Mean (S.D.)
		Absolute	Relative (%)	
Gender	0: Female	356	49.2	51.0 (0.500)
	1: Male	367	50.8	
Marital status	0: Married	312	43.2	0.57 (0.469)
	1: Single	411	56.8	
Driving license	0: Without a driving license	124	17.2	0.83 (0.377)
	1: With a driving license	599	82.8	
Driving experience	0: No experience	123	17.0	2.10 (1.376)
	1: 1–5 year(s)	125	17.3	
	2: 6–10 years	190	26.3	
	3: 11–15 years	128	17.7	
	4: More than 15 years	157	21.7	
Education	2: Below high school diploma	40	5.5	3.10 (1.035)
	3: High school diploma and associate	182	25.2	
	4: Bachelor	214	29.6	
	5: Master	240	33.2	
	6: PhD	47	6.5	
Household size	1	22	3.0	3.60 (1.120)
	2	78	10.8	
	3	245	33.9	
	4	240	33.2	
	5	96	13.3	
	6+	42	5.8	
Household car ownership	0	72	10.0	1.38 (0.809)
	1	373	51.6	
	2	221	30.6	
	3	46	6.4	
	4+	11	1.5	
Income	1: Very low	17	2.4	2.90 (0.796)
	2: Low	201	27.8	
	3: Average	363	50.2	
	4: High	124	17.2	
	5: Very high	18	2.5	

### Measurement model

Numerous studies have shown that the choice of statistical methodology is crucial in research design. Specifically, when the primary goal is to validate and confirm existing theories, structural equation modeling with confirmatory analysis (CB-SEM) is generally recommended. However, when the research focus is on developing new theories and making predictions, a different approach is required. In this case, partial least squares structural equation modeling (PLS-SEM) is often the preferred method^81,82^. As we aimed to validate perceived risk theory, we used AMOS, software that employs the CB-SEM approach. Firstly, a confirmatory factor analysis (CFA) was conducted using Amos V. 24 to examine the psychometric properties of the scale (see Table 3). The fit indices of the model must meet acceptable thresholds to confirm construct validity. Three fit indices were considered for assessing model goodness of fit: the ratio of chi-square value to degrees of freedom (χ^2^/df), the Comparative Fit Index (CFI), and the Root Mean Square Error of Approximation (RMSEA)^83^. Improved model fit is generally indicated by a higher CFI value and a lower value of the other indices. As recommended by Hair et al.^78^, we consider χ^2^/df < 3, CFI > 0.90, and RMSEA < 0.05 as the thresholds for a good fit of our model. Contingent validity can be defined as the agreement between multiple indicators of a single construct. To demonstrate convergent validity, factor loadings should be greater than 0.5. As noted, all the factor loadings ranged from 0.615 to 0.887. Additionally, an Average Variance Extracted (AVE) greater than 0.5 was used as part of the convergent validity assessment, with all AVE values ranging from 0.511 to 0.717. An assessment of discriminant validity considers how much empirical difference exists between the constructs. To achieve discriminant validity^84^, the Fornell-Larcker criterion was applied^85^, suggesting that the square root of the AVE for each construct should be greater than any bivariate correlation involving that construct (see Table 4). Cronbach’s alpha (CA) and composite reliability (CR) were used to assess internal consistency. It is important to note that both CA and CR should be greater than 0.7 to be considered indicative of good internal consistency^85^. According to Table 3, all values of CA and CR were greater than 0.7.

**Table 3:** Measurement model results, convergent validity and internal consistency.

Construct^*^	Item	Loading	*P*-value	CA	CR	AVE
Intention to Use^12,40,84,86^	In the future, I would not hesitate to use an SAVWRS	0.631	0.000	0.847	0.861	0.510
	I intend to use SAVWRS in future on mandatory (e.g., work and educational) trips.	0.781	0.000			
	I intend to use SAVWRS in future at congestion pricing area.	0.691	0.000			
	I intend to use SAVWRS in future at odd-even plate scheme district.	0.695	0.000			
	I intend to use SAVWRS in future on short-distance intracity trips.	0.753	0.000			
	I intend to use SAVWRS in future on long-distance intercity trips.	0.723	0.000			
Trust^23,26,27^	Overall, I can trust SAVWRS	0.861	0.000	0.773	0.756	0.610
	I trust SAVWRS to be safe and reliable in severe weather conditions	0.692	0.000			
Performance Risk^34–36,38,65,69,87^	I am concerned that the safe performance of SAVWRS is not guaranteed.	0.795	0.000	0.700	0.789	0.559
	I am concerned about the lack of proper performance in SAVWRS routing.	0.616	0.000			
	I am concerned about dangers due to misuse of SAVWRS.	0.816	0.000			
Social Risk^29,34,65,69,87,88^	My family doesn’t want me to use an SAVWRS	0.737	0.000	0.704	0.706	0.545
	People dislike SAVWRS; I believe that their opinion is right.	0.740	0.000			
Security Risk^27,34,65,69,87,89^	I am afraid that the data (e.g., position, routes) collected about me during my trips will be stolen	0.835	0.000	0.842	0.883	0.716
	I am concerned that SAVWRS will collect too much personal information from me.	0.824	0.000			
	I am concerned that SAVWRS will use my personal information for other purposes without my authorization.	0.879	0.000			
Financial Risk^34,65,69,87,88^	I am concerned that SAVWRS are too expensive.	0.812	0.000	0.772	0.776	0.634
	I am concerned that SAVWRS will cost more than traditional carsharing services.	0.780	0.000			
Time Risk^34,65,69,87,88^	The learning process to use an SAVWRS might take more time than expected	0.615	0.000	0.779	0.789	0.560
	I am concerned about spending a lot of time to find a car.	0.874	0.000			
	I am worried about the low speed of shared self-driving cars and late arrival at the destination.	0.734	0.000			
Physical Risk^34,65,69,87,88^	I am concerned about sharing SAVWRS with strangers which will lead to physical harm to me.	0.843	0.000	0.770	0.773	0.631
	I am concerned about mental and physical harassment by others in SAVWRS.	0.743	0.000			

* The reference of items has been mentioned at the end of each construct’s name.

CA: Cronbach’s Alpha; CR: Composite Reliability; AVE: Average Variance Extracted.

**Table 4:** Results of discriminant validity test.

	Construct	1	2	3	4	5	6
**1**	Performance	^*^ **0.748**					
**2**	Social	0.693	**0.738**				
**3**	Security	0.634	0.383	**0.846**			
**4**	Financial	0.410	0.183	0.226	**0.796**		
**5**	Time	0.296	0.321	0.142	0.266	**0.748**	
**6**	Physical	0.581	0.496	0.410	0.136	0.181	**0.794**

*Bolded diagonal numbers are the square root of AVE (Average Variance Extracted).

### Structural model (total effect model)

**Fig. 2:**
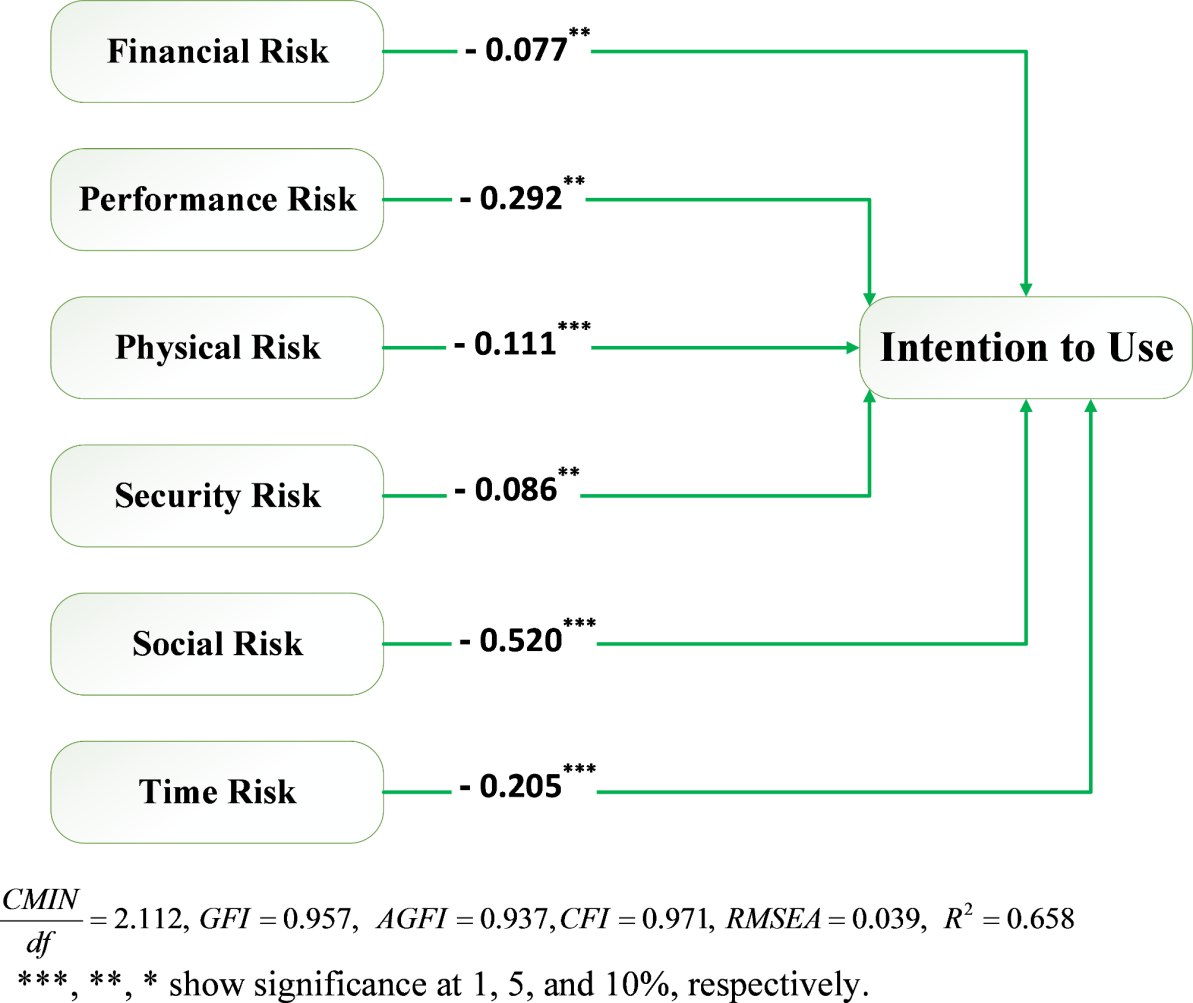
Path coefficients and goodness of fit indices in total effect model (without mediation effect).

An analysis of the hypotheses in the proposed model was carried out using SEM. The proposed model was evaluated according to χ2/df, CFI, and RMSEA goodness-of-fit criteria. In Fig. 2 and Table 5, we present the estimated regression weights. Based on the p-values (lower than 0.05) for all the paths, all the research hypotheses in the total effect model (H1-H6) are accepted, indicating that all the paths are significant. Moreover, the R^2^ value indicates the model’s explanatory power, allowing us to conclude that this research model explained 65.8% of the variance in consumers’ intention to use SAVWRS. The following is a detailed analysis of the results of the structural model.

**Table 5:** Mediation analysis outcome.

Hypothesis	Path	Path coefficient (Total effect model)	Path coefficient (Mediating effect model)	Mediation analysis outcome
H1	FR → BI	− 0.077^**^	− 0.040^*^	Full mediation
H2	PR → BI	− 0.292^**^	− 0.038	Full mediation
H3	PHR → BI	− 0.111^***^	− 0.052	Full mediation
H4	SER → BI	− 0.086^**^	− 0.038	Full mediation
H5	SOR → BI	− 0.520^***^	− 0.385^***^	Partial mediation
H6	TIR → BI	− 0.205^***^	− 0.143^***^	Partial mediation
H7	FR → TR	-	− 0.106^**^	-
H8	PR → TR	-	− 0.889^***^	-
H9	PHR → TR	-	− 0.152^**^	-
H10	SER → TR	-	− 0.123^*^	-
H11	SOR → TR	-	− 0.329^**^	-
H12	TIR → TR		− 0.183^**^	-
H13	TR → BI	-	0.470^***^	-

FR: Financial Risk; PR: Performance Risk; PHR: Physical Risk; SER: Security Risk; SOR: Social Risk; TIR: Time Risk; TR: Trust; BI: Behavioral intention.

***, **, * shows significance at 1, 5, and 10%, respectively.

As illustrated in Fig. 2, of the six hypotheses (H1–H6) in the total effect model, all were supported. Specifically, it can be found that financial risk has a negative impact on consumers’ intention to use SAVWRS (β_H1_ = − 0.077, *p*= 0.029), which supports H1 and is in accordance with Korkmaz et al.’s^38^ findings. In terms performance risk, it is negatively associated with consumers’ intention to use SAVWRS (β_H2_ = − 0.292, *p*= 0.015), which supports H2 and is well-aligned with prior studies^35–39^. It was further found physical risk showed a negative effect on usage intention of SAVWRS (β_H3_ = − 0.111, *p*= 0.006), which supported H3 and is in accordance with Khan et al.’s^36^findings. As found by prior studies^28,35,36,42^, security risk (H4) is also negatively correlated (β_H4_ = − 0.086, *p* = 0.035) with intention to use SAVWRS, indicating a significant effect and support of H4. Social risk as the strongest factor (β_H5_ = − 0.520, *p* < 0.001) of consumers’ usage intention of SAVWRS, which supports H5. Finally, it can be found that time risk has a negative impact on consumers’ intention to use SAVWRS (β_H6_ = − 0.205, *p* < 0.001), which supports H6.

### Trust, as a mediator (mediating effect model)

**Fig. 3:**
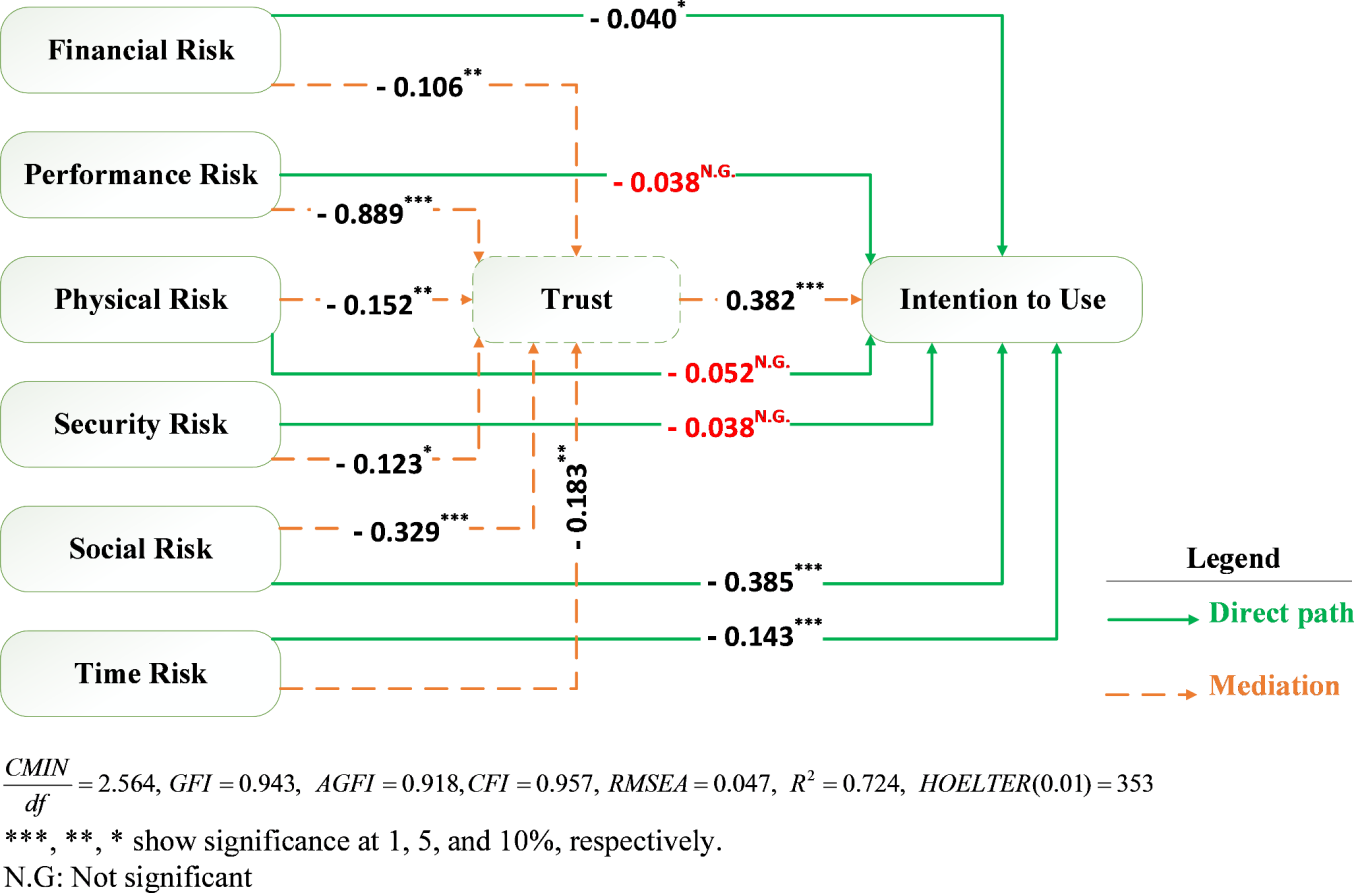
Path coefficients and goodness of fit indices in mediating effect model.

According to the research objectives, we have examined the mediating effect of trust in the relationship between perceived risk and usage intention. A mediation variable (also known as a mediator) plays a crucial role in explaining how or why of an observed relationship exists between an IV and its DV. Instead of assuming a direct causal relationship between the IV and DV (a common oversimplification), a mediation model proposes that the IV influences the mediator, which in turn influences the DV. In other words, when there is a significant mediator between two variables, it implies that there is an intermediate variable that plays a crucial role in explaining the relationship between the IV and the DV^78^. To test mediation hypotheses, Baron and Kenny^90^method was utilized. This approach tests mediation using three regression analyses: (1) The IV’s prediction of the DV (total effect model which encompasses only the direct pathways without the mediator, i.e., H1-H6); (2) The IV’s prediction of the mediator; and (3) The prediction of the DV by both the IV and the mediator (mediating effect model, i.e., H1-H13). To support mediation, the results must meet the following conditions: (1) It must be proved that the IV influences the DV in a significant manner in the first regression equation (Total effect model); (2) In the second regression equation, the IV significantly influences the mediator; and (3) In the third equation, the mediator must have a significant impact on the DV. It is considered to be fully mediator when all the above conditions are met and the IV no longer influences the DV. Moreover, partial mediation implies a direct relationship between the IV and DVs in addition to a significant relationship between the mediator and the DV^78^. Since all the regression coefficients in the total effect model (Fig. 2) are significant, in the next step, trust was added to the model as a mediator, and we assessed the mediating effect of trust in the mediating effect model presented in Table 5; Fig. 3.

The mediation analysis (Table 5) shows that trust fully mediates the negative and significant relationship between performance risk and intention to use. Regarding the negative and significant relationship between security risk and intention to use, trust also has a fully mediating effect, which is in accordance with findings of Waung et al^28^. and Zhang et al^27^. Due to the lack of a steering wheel and human control over the vehicle in SAVWRS, individuals’ concerns about physical injuries fully mediate their intention to use this technology through trust. This full mediating effect is also observed in the relationship between financial risk and intention to use. Additionally, trust has a partial mediating effect on the negative and significant relationship between social risk and intention to use SAVWRS. Furthermore, a direct effect of social risk on intention to use is still observable as a result of the partially mediating effect of trust. Lastly, our findings reveal that the relationship between time risk and intention to use is not direct. Instead, there is a partial mediating effect, indicating that the influence of time risk on intention to use is partially explained by trust.

### Multi-group moderation analysis

The relationships between perceived risk dimensions, trust, and intention to use SAVWRS have been analyzed through a multi-group moderation analysis^1^ (corresponding to moderation analysis for categorical moderator variables), to examine the moderating effect of generation (Table 6). Multi-group analysis results in separate models for each group, enabling researchers compare the model parameters across different groups to understand how the relationships differ. Moreover, the critical ratio for difference (CRD) has been utilized to check for any significant heterogeneity among millennials and baby boomers. Based on the CRD values, it can be concluded that there is significant heterogeneity among the millennial and baby boomer generations in their intention to use SAVWRS. This indicates that the two generations have different attitudes and preferences regarding SAVWRS. In terms of trust in SAVWRS, baby boomers are more sensitive to performance risk, security risk, and financial risk, which aligns well with the findings of Korkmaz et al^38^. While millennials are more sensitive to social and physical risks. Moreover, it can be observed that time risk plays a more critical role in the usage intention of millennials rather than baby boomers, which may be due to their busier schedules; however, the difference is not statistically significant. In greater detail, the results of the multi-group moderation analysis indicate that performance risk has a more pronounced impact on trust formation among baby boomers than among millennials^91,92^. Similarly, security risk and financial risk exhibit a stronger influence on trust formation for baby boomers compared to millennials, while social risk and physical risk play a more significant role in trust formation among millennials relative to baby boomers. Therefore, it can be concluded that baby boomers have a more traditional approach to SAVWRS, while millennials are more open to the new possibilities and potential of SAVWRS due to their greater familiarity with technology^35,36^.

**Table 6:** Multi-group moderation analysis of generation.

Hypothesis	Casual paths	Millennial		Baby Boomer		CRD	Result
		Estimate	*P*-value	Estimate	*P*-value		
H14	PR → TR	− 1.352	0.000	− 1.692	0.000	− 8.892***	Supported
H15	SOR → TR	− 0.235	0.000	− 0.012	0.594	5.812***	Supported
H16	SER → TR	− 0.274	0.000	− 0.376	0.000	− 3.351***	Supported
H17	FR → TR	− 0.216	0.000	− 0.318	0.000	− 3.103***	Supported
H18	PHR → TR	− 0.295	0.000	− 0.173	0.000	3.351***	Supported
H19	TIR → TR	− 0.175	0.000	− 0.153	0.000	− 0.372	Rejected
H20	TR → BI	0.478	0.000	0.463	0.000	− 0.119	Rejected

FR: Financial Risk; PR: Performance Risk; PHR: Physical Risk; SER: Security Risk; SOR: Social Risk; TIR: Time Risk; TR: Trust; BI: Behavioral intention.

***, **, * shows significance at 1, 5, and 10%, respectively.

## Discussion

### Theoretical implications

In this study, it is hypothesized that different dimensions of perceived risk may contribute to consumers’ hesitation to use SAVWRS. We investigate the mediating effect of trust and the moderating effect of generation to gain a deeper understanding of the relationship between perceived risk and usage intention of SAVWRS. The insights obtained from this study regarding the relation between consumer intention and perceived risk under trust enrich existing research on consumer behavior in the sharing and autonomous economy from the perspective of risk.

Based on the estimated coefficients of the total effect model (Table 5), it can be seen that social risk is the strongest factor hindering respondents’ usage intention. It is likely due to the fact that in most developing countries, there is a trend of social prestige toward car ownership^93^. The second strongest factor is performance risk, indicating that respondents are concerned about the performance of these technology services which is in accordance with previous studies^26,42^. Time risk is the third strongest factor, indicating that people prioritize travel modes that offer the shortest travel time. This is especially true in heavily congested cities such as Tehran, where fast and reliable transportation is even more needed^94^. Physical and security risks are the fourth and fifth important influential factors hindering respondents’ intention to use. Moreover, financial risk is the least important factor that negatively affects respondents’ usage intention. High costs associated with travel reduce the overall demand for a certain transportation mode, resulting in lower usage intention. This is especially true in cities where public transportation is already expensive and not widely available^86^. Finally, when it comes to individuals’ trust, it can be seen that performance risk is the most critical factor affecting trust in SAVWRS.

### Practical implications

A number of recommendations are suggested for policymakers and SAV service providers on how to promote the popularity of SAV services based on the empirical findings of this study. Security risk is a significant factor negatively associated with the usage intention of SAVWRS. As an implication, it is recommended that governments take measures to ensure that service providers safeguard the personal information of citizens to alleviate privacy concerns. Moreover, policymakers should adopt measures to enhance control over how service providers manage and use private information. In addition, the implementation of advanced security protocols, including strong encryption to ensure data confidentiality and user-centric functionalities that enable riders to share their trip information with designated contacts, can mitigate perceived security risk. Furthermore, conducting rigorous stress tests and providing transparent reports of safety incidents can enhance consumer trust. In terms of social risk, which reflects the impact of the surrounding environment, a suitable strategy to encourage the use of SAVWRS is to advertise across all possible media channels, such as social media and mass media to portray a positive image of using this shared service. Policymakers should inform the public about the wide range of benefits that SAVWRS provides, such as reducing congestion, travel costs, fuel consumption, and pollutants. When addressing physical risk, it is recommended to provide special services. For example, the use of closed-circuit televisions (CCTVs) enhances the feeling of security among passengers^95,96^, which in turn increases their willingness to use the service and reduces their worries about harassment. Providing information about how a vehicle would protect passengers in the event of critical system failures is also helpful in reducing potential users’ performance concerns. If possible, manufacturers should make their safety algorithms and data publicly available for review. In addition, policymakers should collaborate closely with academics and manufacturers in order to establish safety regulations and ensure that safety standards are strictly followed by developers^27^. Since trust influences users’ intention, it is essential for authorities to build trust among SAVs’ users. It is therefore imperative to ensure the safety of this technology. Continuous monitoring of SAVs, regular checks to prevent potential defects, and the presence of cameras and operators inside the vehicle can help reduce concerns and foster trust. Furthermore, it is recommended to develop a trust-building framework. It is further recommended to develop a trust-building framework. To ensure the transparency and reliability of SAVWRS systems, policymakers must establish comprehensive regulatory frameworks. These frameworks should encompass rigorous safety standards, continuous performance evaluations, and clear operational guidelines. Disseminating detailed and accessible information about the technological capabilities and safety protocols of these vehicles is crucial for bolstering public trust and confidence. To reduce the time risks associated with SAVs, policymakers and service providers should establish dedicated lanes, utilize large fleets, and implement dynamic ride-sharing algorithms in their software to expedite the matching process for SAVWRS. Considering the significant differences between generations, it is recommended that policymakers and governments to hold educational campaigns. Public awareness campaigns should aim to elucidate the complexities of autonomous technologies while addressing the unique concerns of different demographic groups. For Baby Boomers, highlighting the enduring values of safety and reliability may prove effective, while Millennials are likely to be more receptive to messages that emphasize innovation, economic advantages, and environmental sustainability. To reduce individuals’ perception of financial risk associated with such services, it is recommended that policymakers create regulations ensuring fair and transparent pricing. Moreover, offering incentives such as loyalty programs or subsidizing rides for specific socio-demographics, particularly for low-income individuals or environmentally conscious consumers, can encourage people to use these services. Additionally, developing an application for addressing with users’ complaints can play a vital role in enhancing usage intention. Finally, it is imperative to ensure that SAVWRS services operate within the framework of the law.

### Limitations and future research

By addressing previously identified gaps, the findings of this research significantly contribute to a deeper understanding of the impact of perceived risk dimensions on the usage intention of SAVWRS. Despite the interesting findings and implications of our research, there are some limitations. First, respondents did not have any actual experience with SAVs; their preferences were based on what they knew about SAVs from mass and social media. As users become more familiar with the system and gain a better understanding of how it operates, the levels of usage intention and perceived risk are expected to change in the future. Therefore, it is recommended that longitudinal studies be conducted to further understand the impact of diffusion on user acceptance after users have had more interactive experiences with such services. Another limitation is that we measured trust using a one-dimensional construct. It is recommended to investigate the impact of other dimensions of trust, such as benevolence, integrity, and competence. Moreover, exploring the impact of the five major personality traits on respondents’ perceived risk and usage intention is also recommended. Finally, we used a SEM model to investigate the effects of various latent factors on the intention to use SAVWRS. However, it is suggested to employ the integrated choice and latent variable (ICLV) model to investigate the simultaneous effects of latent factors, socio-economic characteristics, and travel-related characteristics.

## Data Availability

The datasets generated during and analyzed during the current study are not publicly available due to restrictions e.g. privacy or ethical but are available from the corresponding author on reasonable request.

## Footnotes

1 Before the moderation analysis, ANOVA was conducted to explore the impact of the “Generation” type (e.g., millennials, baby boomers) on the intention to use shared autonomous vehicles. The results revealed a significant effect of “Generation” type (partial η² = 0.024, p-value = 0.001, F = 12.545). In other words, the comparison of intention to use autonomous vehicles across different generations indicates a statistically significant difference.
